# Association of Serum Visfatin Levels with Coronary Artery Disease and Biochemical Parameters in Patients Undergoing Maintenance Hemodialysis

**DOI:** 10.3390/jcm15208042

**Published:** 2026-10-16

**Authors:** Nimet Aktas, Mustafa Gullulu, Nazife Nur Ozer Sensoy, Alparslan Ersoy, Baris Sensoy

**Affiliations:** 1 Department of Nephrology, University of Health Sciences, Bursa Yüksek İhtisas Training and Research Hospital, 16310 Bursa, Türkiye; nurozer_@hotmail.com; 2 Department of Nephrology, Faculty of Medicine, Bursa Uludağ University, 16059 Bursa, Türkiye; mgullulu@uludag.edu.tr (M.G.); alpersoy@uludag.edu.tr (A.E.); 3 Department of Cardiology, University of Health Sciences, Bursa Yüksek İhtisas Training and Research Hospital, 16059 Bursa, Türkiye; gs2000bs@yahoo.com

**Keywords:** visfatin, hemodialysis, coronary artery disease, adipokines, chronic kidney disease, biomarkers

## Abstract

**Background**: Cardiovascular disease is a major cause of morbidity and mortality in patients with end-stage kidney disease receiving maintenance hemodialysis. Visfatin is a pro-inflammatory adipokine involved in metabolic and inflammatory pathways; however, its relationship with established coronary artery disease (CAD) in hemodialysis patients remains uncertain. This study evaluated the associations of serum visfatin concentrations with hemodialysis status, clinically documented CAD, and selected metabolic and biochemical parameters. **Methods**: This cross-sectional study included 130 non-diabetic adults classified into four groups: healthy controls (n = 40), participants with preserved renal function and established CAD (n = 28), maintenance hemodialysis patients without CAD (n = 30), and maintenance hemodialysis patients with established CAD (n = 32). Demographic, anthropometric, and laboratory variables were recorded. Serum visfatin concentrations were measured using an enzyme-linked immunosorbent assay. Between-group comparisons and exploratory correlation analyses were performed. Analysis of covariance was additionally used to assess between-group differences in visfatin after adjustment for age, sex, hypertension, and BMI, with body fat percentage substituted for BMI in a sensitivity analysis. **Results**: Median serum visfatin concentration was higher in the pooled hemodialysis groups than in the pooled non-hemodialysis groups (35.75 vs. 30.51 ng/mL, *p* = 0.014). In unadjusted pairwise comparisons, healthy controls had lower visfatin concentrations than participants with preserved renal function and CAD (*p* = 0.007), hemodialysis patients without CAD (*p* = 0.001), and hemodialysis patients with CAD (*p* = 0.004). However, visfatin concentrations did not differ between hemodialysis patients with and without established CAD (*p* = 0.972). After adjustment for age, sex, hypertension, and BMI, the overall between-group effect was no longer statistically significant (F(3, 122)= 2.054, *p* = 0.110, partial η^2^ = 0.048). The result was similar in the sensitivity analysis replacing BMI with body fat percentage (*p* = 0.060). In exploratory analyses of the pooled hemodialysis population, visfatin was positively correlated with fasting insulin (r = 0.269, *p* = 0.039), HOMA-IR (r = 0.277, *p* = 0.033), and serum uric acid (r = 0.263, *p* = 0.039). **Conclusions**: Higher serum visfatin concentrations were observed in patients receiving maintenance hemodialysis in unadjusted analyses, but the overall between-group difference was attenuated after adjustment for demographic and body-composition variables. Visfatin did not distinguish hemodialysis patients according to established CAD status. These findings do not demonstrate an independent association of circulating visfatin with either hemodialysis status or clinically documented CAD. Given the cross-sectional design and exploratory nature of the correlation analyses, further prospective studies with larger samples and standardized assessment of coronary disease burden are warranted.

## 1. Introduction

Cardiovascular disease is the leading cause of morbidity and mortality in patients with chronic kidney disease, particularly among those with end-stage kidney disease receiving maintenance hemodialysis [1,2,3]. Cardiovascular mortality in this population is substantially higher than in the general population, and the increased risk is evident across all stages of kidney disease [4]. Traditional cardiovascular risk factors alone do not fully explain this excessive burden. Uremia-related nontraditional factors, including chronic inflammation, oxidative stress, endothelial dysfunction, anemia, abnormalities of mineral metabolism, and malnutrition, may also contribute to cardiovascular disease in hemodialysis patients [5,6].

Adipose tissue is now recognized as an active endocrine and immune organ that secretes several biologically active molecules known as adipokines. These mediators are involved in metabolic regulation, inflammation, insulin resistance, and vascular homeostasis [7]. In patients with chronic kidney disease, altered adipokine concentrations have also been associated with nutritional status, glucose metabolism, endothelial dysfunction, and cardiovascular disease [7,8]. Visfatin, also known as extracellular nicotinamide phosphoribosyltransferase, is a pleiotropic protein with metabolic and pro-inflammatory properties. Experimental studies have suggested potential cardioprotective effects [9,10], whereas clinical studies have reported associations between higher circulating visfatin concentrations and endothelial dysfunction or atherosclerosis-related parameters in patients with kidney disease [11,12].

Despite these observations, it remains uncertain whether increased circulating visfatin in hemodialysis patients primarily reflects impaired kidney function, metabolic and inflammatory disturbances, or coronary artery disease. Moreover, relatively few studies have evaluated circulating visfatin using parallel comparison groups defined simultaneously by kidney function and clinically documented coronary artery disease status [13]. Therefore, this study aimed to compare serum visfatin concentrations among four non-diabetic groups comprising individuals with preserved kidney function and hemodialysis patients, with and without coronary artery disease. We also examined the associations of serum visfatin with selected anthropometric, metabolic, inflammatory, and biochemical parameters.

This study contributes to the existing literature in several ways. First, it evaluates circulating visfatin across four parallel groups defined simultaneously by kidney function and clinically documented CAD status, allowing the potential influences of hemodialysis and CAD to be examined within the same study framework. Second, by restricting the cohort to non-diabetic participants, the study reduces the potential confounding effect of diabetes on circulating visfatin concentrations. Third, the analysis incorporates adjustment for important demographic and body-composition differences and explores whether visfatin distinguishes hemodialysis patients with and without clinically documented CAD. Finally, the study provides additional data on the metabolic and biochemical correlates of visfatin in maintenance hemodialysis patients. Together, these features address an area in which comparative clinical data remain limited and help clarify the potential, as well as the limitations, of circulating visfatin as a cardiovascular biomarker in this population.

## 2. Materials and Methods

### 2.1. Study Design and Participants

This cross-sectional study was approved by the Ethics Committee of Uludağ University Faculty of Medicine (Approval No. 2010-8/2, dated 2 September 2010). All participants provided written informed consent before enrollment.

Participants were recruited between April and November 2011. A total of 130 non-diabetic adults were classified into four groups according to kidney function and coronary artery disease (CAD) status: healthy controls (HC, n = 40), participants with preserved kidney function and established CAD (CAD controls, n = 28), maintenance hemodialysis patients without clinically documented CAD (HD, n = 30), and maintenance hemodialysis patients with established CAD (HD-CAD, n = 32). Healthy controls were recruited from the outpatient clinics of the Department of Internal Medicine. CAD controls were recruited from among patients undergoing coronary angiography at the Cardiology Hemodynamics Laboratory. Hemodialysis patients were recruited from the Hemodialysis Unit of Uludağ University Faculty of Medicine and participating dialysis centers in Bursa, Türkiye.

### 2.2. Eligibility Criteria

Participants aged 18 years or older were eligible for inclusion. Diabetes mellitus was excluded from all study groups because of its potential influence on circulating visfatin concentrations. Additional exclusion criteria included active infection, malignancy, chronic liver disease, autoimmune or inflammatory disorders, and current treatment with systemic corticosteroids or growth hormone.

Maintenance hemodialysis patients were required to have been receiving three-times-weekly hemodialysis for at least six months before enrollment. Healthy controls were required to have no history or clinical evidence of kidney disease or CAD and a serum creatinine concentration ≤1.2 mg/dL. Participants in the CAD control group were required to have established CAD, preserved kidney function, and a serum creatinine concentration ≤1.2 mg/dL.

### 2.3. Clinical Assessment

Demographic characteristics, smoking status, medical history, dialysis vintage, and cardiovascular risk factors were recorded for all participants. Physical examination, blood pressure measurement, electrocardiography, and anthropometric assessments, including body weight, height, body mass index (BMI), waist circumference, hip circumference, and body fat percentage, were performed using standardized methods.

CAD status was determined from medical history, clinical assessment, electrocardiographic findings, and available coronary angiography reports. Established CAD was defined by the presence of at least one of the following: a documented history of myocardial infarction, previous percutaneous coronary intervention with stent implantation, previous coronary artery bypass grafting, or coronary artery stenosis of at least 40% demonstrated by coronary angiography. The ≥40% threshold was used to identify angiographically documented coronary atherosclerotic disease and was not intended to represent a threshold for hemodynamically significant or revascularization-requiring stenosis. Coronary imaging was not systematically performed in participants classified as being without CAD; therefore, these groups should be interpreted as having no clinically documented CAD rather than as having angiographically excluded coronary atherosclerosis.

Participants assigned to the groups without clinically documented CAD had no history of CAD or other cardiovascular disease, no ischemic symptoms, no relevant abnormalities on cardiovascular examination, and no pathological findings on electrocardiography. Coronary imaging was not systematically performed in the HD group without clinically documented CAD.

### 2.4. Laboratory Measurements

Venous blood samples were collected after an overnight fast of 8–12 h. In the control groups, blood sampling was performed between 08:30 and 10:00 a.m. In hemodialysis patients, samples were obtained immediately before the dialysis session following the longest interdialytic interval. Serum samples collected for visfatin measurement were separated immediately and stored at −80 °C until analysis. All stored samples were analyzed in a single batch.

Routine laboratory measurements included complete blood count, fasting plasma glucose, fasting insulin, urea, creatinine, uric acid, calcium, phosphorus, alkaline phosphatase, albumin, total protein, lipid profile, iron parameters, ferritin, parathyroid hormone, and C-reactive protein. Complete blood counts were measured using an automated hematology analyzer (Sysmex XT-1000, Sysmex Corporation, Kobe, Japan). Routine biochemical parameters were measured using photometric methods on an ARCHITECT c16000 analyzer (Abbott Diagnostics, Abbott Park, IL, USA). Serum ferritin and parathyroid hormone concentrations were determined by chemiluminescent microparticle immunoassay using an ARCHITECT i2000 analyzer (Abbott Diagnostics). Serum insulin was measured by electrochemiluminescence immunoassay using a Cobas e 411 analyzer (Roche Diagnostics, Mannheim, Germany), and C-reactive protein was measured by immunonephelometry using a Siemens CardioPhase assay.

Serum visfatin concentrations were measured using a commercially available Visfatin C-terminal (Human) enzyme immunoassay kit (Catalog No. EK-003-80; Phoenix Pharmaceuticals, Belmont, CA, USA) according to the manufacturer’s instructions. According to the manufacturer’s specifications, the analytical sensitivity was 2.3 ng/mL, with intra-assay and inter-assay coefficients of variation of <10% and <15%, respectively, and a linear range of 2.3–40 ng/mL. The assay is directed against the C-terminal region of human visfatin and also recognizes recombinant full-length human visfatin. For descriptive analyses, visfatin concentrations were categorized as 2.3–40 ng/mL or >40 ng/mL based on the manufacturer-provided assay range. This range was not considered a clinically validated diagnostic cutoff and was used solely for exploratory descriptive analyses. Insulin resistance was estimated using the Homeostatic Model Assessment of Insulin Resistance (HOMA-IR), calculated as fasting insulin (μU/mL) × fasting plasma glucose (mmol/L)/22.5. Glucose concentrations measured in mg/dL were converted to mmol/L before calculation.

eKt/V, equilibrated Kt/V; URR, urea reduction ratio. Blood samples were obtained immediately before the dialysis session following the longest interdialytic interval.

### 2.5. Statistical Analysis

Statistical analyses were performed using SPSS Statistics version 13.0 (SPSS Inc., Chicago, IL, USA). The distribution of continuous variables was assessed using the Shapiro–Wilk test. Normally distributed continuous variables are presented as mean ± standard deviation, whereas non-normally distributed variables are presented as median (minimum–maximum). For non-normally distributed variables, comparisons between two groups were performed using the Mann–Whitney U test, and comparisons involving more than two groups were performed using the Kruskal–Wallis test. When the Kruskal–Wallis test indicated an overall group difference, unadjusted pairwise comparisons were conducted using the Mann–Whitney U test. These post hoc comparisons were considered exploratory. Associations between continuous variables were evaluated using Pearson correlation coefficients for approximately normally distributed variables with linear relationships and Spearman rank correlation coefficients for non-normally distributed variables or when parametric assumptions were not met. Categorical variables were compared using the Pearson chi-square test or Fisher’s exact test, as appropriate. Statistical significance was defined as *p* < 0.05. To account for baseline differences between groups, analysis of covariance (ANCOVA) was additionally performed with serum visfatin concentration as the dependent variable, study group, sex, and hypertension as fixed factors, and age and BMI as covariates. A sensitivity analysis was performed by replacing BMI with body fat percentage. Homogeneity of regression slopes was assessed by testing group-by-covariate interaction terms. Bonferroni adjustment was applied to pairwise comparisons of estimated marginal means. A post hoc power analysis and sensitivity analysis for the primary HD versus HD-CAD comparison were performed using G*Power version 3.1.9.7.

## 3. Results

### 3.1. Participant Characteristics

A total of 130 participants were included: 40 healthy controls, 28 CAD controls with preserved kidney function, 30 maintenance hemodialysis patients without clinically documented CAD, and 32 maintenance hemodialysis patients with established CAD.

Demographic, clinical, and anthropometric characteristics are presented in Table 1. Age differed significantly among the four groups and was lowest in healthy controls and highest in the HD-CAD group (*p* < 0.001). Sex distribution also differed, with the highest proportion of women in the healthy control group (*p* = 0.005). The prevalence of hypertension increased from 15.0% in healthy controls to 78.1% in the HD-CAD group (*p* < 0.001).

Systolic blood pressure differed among the groups and was highest in the HD group (*p* = 0.003), whereas diastolic blood pressure was comparable (*p* = 0.646). BMI also differed across the groups (*p* = 0.003), while waist circumference was similar (*p* = 0.988). Among patients receiving hemodialysis, dialysis duration did not differ between those with and without CAD (*p* = 0.459). Body weight and body fat percentage also differed significantly among the four groups (*p* = 0.005 and *p* = 0.008, respectively), with the highest values observed in healthy controls.

### 3.2. Laboratory Findings

Laboratory findings are summarized in Table 2. Fasting glucose, insulin, and HOMA-IR differed significantly among the four groups, with the highest median values observed in the HD-CAD group (all *p* < 0.001). Urea and creatinine concentrations were substantially higher in both hemodialysis groups than in the groups with preserved kidney function. Uric acid, calcium, phosphorus, albumin, hemoglobin, CRP, and PTH concentrations also differed significantly across the groups (all *p* < 0.001). Among hemodialysis participants, dialysis adequacy did not differ significantly according to CAD status. Median Kt/V was 1.54 (1.01–2.38) in the HD group and 1.44 (1.03–2.17) in the HD-CAD group (*p* = 0.192), while median URR was 72.36% (63.71–85.28) and 74.88% (63.22–83.19), respectively (*p* = 0.667).

Lipid parameters differed among the four groups (all *p* < 0.001). Healthy controls had the highest median total and LDL cholesterol concentrations, whereas the HD-CAD group had the lowest median HDL cholesterol and the highest median triglyceride concentration.

### 3.3. Serum Visfatin Concentrations

When participants were pooled according to kidney function, recalculated median serum visfatin concentrations were 35.75 ng/mL in the combined hemodialysis group and 30.51 ng/mL in participants with preserved kidney function (*p* = 0.014).

Across the four individual groups, median visfatin concentrations were 26.52 ng/mL in healthy controls, 33.68 ng/mL in CAD controls, 35.96 ng/mL in the HD group, and 35.74 ng/mL in the HD-CAD group (Table 3). Pairwise comparisons demonstrated lower visfatin concentrations in healthy controls than in CAD controls (*p* = 0.007), HD patients (*p* = 0.001), and HD-CAD patients (*p* = 0.004). Visfatin concentrations were comparable among CAD controls, HD patients, and HD-CAD patients (all *p* ≥ 0.773). In particular, no difference was observed between hemodialysis patients with and without CAD (*p* = 0.972).

In an exploratory analysis using the manufacturer-provided threshold of 40 ng/mL, no significant differences were observed in the evaluated clinical or laboratory parameters between participants with serum visfatin concentrations of 2.3–40 ng/mL and those with concentrations > 40 ng/mL.

In the adjusted ANCOVA model including age, sex, hypertension, and BMI, the overall effect of study group on serum visfatin concentration was no longer statistically significant (F (3, 122) = 2.054, *p* = 0.110, partial η^2^ = 0.048). The adjusted estimated marginal means (95% CI) were 28.40 (24.84–31.95) ng/mL in healthy controls, 33.60 (29.61–37.59) ng/mL in CAD controls, 34.49 (30.84–38.14) ng/mL in HD patients, and 33.75 (30.00–37.51) ng/mL in HD-CAD patients. Bonferroni-adjusted pairwise comparisons were not statistically significant, including the comparison between HD and HD-CAD patients (mean difference, 0.74 ng/mL; adjusted *p* = 1.000). In a sensitivity analysis replacing BMI with body fat percentage, the overall group effect remained non-significant (F(3, 122) = 2.537, *p* = 0.060, partial η^2^ = 0.059) presented in Table 4.

In an exploratory analysis using the manufacturer-provided assay range, 106 participants had serum visfatin concentrations of 2.3–40 ng/mL and 24 had concentrations > 40 ng/mL. This categorization was used for descriptive purposes only and was not considered a clinically validated diagnostic threshold.

### 3.4. Correlations of Serum Visfatin

In the combined hemodialysis group, serum visfatin was positively correlated with fasting insulin (r = 0.269, *p* = 0.039), HOMA-IR (r = 0.277, *p* = 0.033), and uric acid (r = 0.263, *p* = 0.039). Among participants with preserved kidney function, visfatin was positively correlated with urea (r = 0.275, *p* = 0.023), creatinine (r = 0.285, *p* = 0.018), uric acid (r = 0.250, *p* = 0.040), and PTH (r = 0.262, *p* = 0.031), and inversely correlated with total cholesterol (r = −0.246, *p* = 0.043) and triglycerides (r = −0.259, *p* = 0.033).

Selected group-specific correlations are presented in Table 5. In healthy controls, visfatin was inversely correlated with triglycerides (r = −0.359, *p* = 0.023). In CAD controls, visfatin was positively correlated with systolic blood pressure (r = 0.496, *p* = 0.007), urea (r = 0.469, *p* = 0.012), and creatinine (r = 0.381, *p* = 0.045). In HD patients without clinically documented CAD, visfatin was positively correlated with uric acid (r = 0.394, *p* = 0.031). No nominally significant correlations were identified in the HD-CAD group. No group-specific associations were observed between visfatin and age, BMI, waist circumference, or CRP. All correlation analyses were exploratory, and *p* values were not adjusted for multiple testing.

## 4. Discussion

The principal finding was that serum visfatin concentrations differed among the study groups in unadjusted analyses, with the lowest median concentrations observed in healthy controls. However, after adjustment for age, sex, hypertension, and BMI, the overall between-group difference was no longer statistically significant. This finding was consistent in a sensitivity analysis in which BMI was replaced by body fat percentage. In addition, no significant difference was observed between hemodialysis patients with and without clinically documented CAD in either unadjusted or adjusted analyses. Several relationships with metabolic and biochemical variables were also identified, although these analyses were exploratory. Taken together, these findings suggest that the observed unadjusted differences in circulating visfatin may be partly influenced by baseline demographic and body-composition differences and do not establish an independent association between visfatin and hemodialysis or CAD status.

Previous studies indicate that circulating visfatin is altered in advanced kidney disease, although its biological and clinical significance remains uncertain. Nüsken et al. reported increased concentrations of biologically active visfatin in patients receiving maintenance hemodialysis [14]. Other CKD studies have linked circulating visfatin with kidney function, appetite, endothelial function, and several nutritional and metabolic variables [11,15,16]. The adipokine profile of hemodialysis patients may be influenced by the combined effects of impaired kidney function, chronic inflammation, altered body composition, and disturbances in energy metabolism [8,17,18]. In the present study, higher visfatin concentrations were observed in the pooled hemodialysis population in unadjusted analyses; however, the overall between-group effect was attenuated after adjustment for demographic and body-composition differences. Accordingly, the observed pattern cannot be attributed specifically to kidney failure itself. Moreover, the present study did not evaluate visfatin clearance, tissue production, biological activity, or longitudinal changes, and therefore cannot establish a specific underlying mechanism. The higher circulating visfatin concentrations observed in hemodialysis patients may therefore reflect several overlapping mechanisms, including reduced renal clearance, chronic inflammation, altered adipose tissue signaling, and metabolic disturbances associated with advanced kidney failure and dialysis treatment.

Evidence concerning the relationship between visfatin and coronary atherosclerosis has been inconsistent [13,19]. Higher circulating concentrations have been reported in patients with coronary heart disease, and associations with endothelial dysfunction, vascular or valvular calcification, and other atherosclerotic measures have been described in selected populations [12,13,20]. In the present study, however, visfatin concentrations did not differ between hemodialysis patients with and without established CAD. Values were also comparable among CAD controls and both hemodialysis groups. After adjustment for age, sex, hypertension, and BMI, no statistically significant overall between-group difference in visfatin remained, and the adjusted comparison between HD and HD-CAD patients was also non-significant. These findings therefore do not provide evidence that circulating visfatin differentiates patients according to clinically documented CAD status in this cohort. Nevertheless, the absence of a between-group difference should not be interpreted as evidence that visfatin has no role in vascular disease. The relatively modest sample size limits the ability to exclude smaller clinically relevant differences. In addition, coronary imaging was not systematically performed in participants classified as having no CAD; therefore, subclinical or previously unrecognized coronary disease may have resulted in some degree of misclassification.

Recent studies have continued to examine the cardiovascular context of visfatin in kidney failure. In a small case–control study, serum visfatin levels and peripheral blood mononuclear cell NAMPT expression were highest among patients receiving hemodialysis [21]. Two subsequent cross-sectional studies reported associations between higher serum visfatin levels and coronary artery calcification in maintenance hemodialysis patients [22] and cardiac valve calcification in hemodialysis patients [20]. However, these studies evaluated imaging-defined calcification rather than clinically established coronary artery disease and therefore do not establish causality. Accordingly, they address related but distinct cardiovascular phenotypes and are not directly comparable with our analysis based on clinically documented CAD. Importantly, the absence of a significant association between visfatin and CAD status in our adjusted analyses does not exclude a potential relationship with subclinical vascular or valvular calcification.

The metabolic correlates of visfatin also remain uncertain. In the combined hemodialysis population, visfatin showed modest positive correlations with fasting insulin, HOMA-IR, and uric acid. Previous studies have reported variable relationships between circulating visfatin and insulin sensitivity, glucose metabolism, adiposity, and lipid parameters [15,16,23,24]. An inverse association with HDL cholesterol has also been described in maintenance hemodialysis patients [14], although this relationship was not observed in our group-specific analyses. The correlations identified in the present study were small, exploratory, and unadjusted for multiple testing. HOMA-IR is an indirect surrogate of insulin resistance and should be interpreted cautiously in patients with advanced kidney failure, in whom altered insulin metabolism and clearance may affect fasting insulin-based indices. Moreover, all participants with diabetes were excluded. Accordingly, the observed correlations should be regarded as hypothesis-generating rather than evidence of a causal or clinically validated relationship between visfatin and insulin resistance.

Several limitations should be considered when interpreting these findings. First, the cross-sectional design does not establish whether changes in visfatin preceded or followed kidney failure, metabolic abnormalities, or CAD. Second, the study groups were not matched and differed in age, sex distribution, body weight, body mass index, and body fat percentage. We therefore performed adjusted analyses accounting for age, sex, hypertension, and BMI, together with a sensitivity analysis replacing BMI with body fat percentage. Although the overall group effect was no longer statistically significant in either adjusted model, residual confounding from measured and unmeasured factors cannot be excluded. Third, CAD was confirmed from clinical history, previous coronary procedures, or available angiographic findings, whereas coronary angiography was not systematically performed in participants classified as being without CAD. Detailed dialysis prescription characteristics, including dialyzer membrane type and blood and dialysate flow rates, as well as comprehensive medication data potentially affecting metabolic and inflammatory parameters, were not systematically available and therefore could not be incorporated into the analyses. Some degree of disease misclassification is therefore possible. Fourth, visfatin was measured at a single time point, and the manufacturer-provided concentration range was not a clinically validated diagnostic threshold. Bonferroni correction was applied to the primary pairwise comparisons of visfatin concentrations; however, the correlation analyses were exploratory and were not adjusted for multiplicity. Finally, the modest sample size, exclusion of patients with diabetes, and absence of prospective cardiovascular outcomes limit both the precision and generalizability of the results. The post hoc analysis for the HD versus HD-CAD comparison indicated low achieved power for the observed effect, while a power sensitivity analysis showed that the available sample sizes would provide 80% power only for an effect size of approximately Cohen’s d = 0.72. Accordingly, smaller but potentially clinically relevant differences in visfatin concentrations between HD patients with and without CAD cannot be excluded.

The study also has several methodological strengths. The four-group design permitted parallel comparisons according to both kidney-function and CAD status. Exclusion of participants with diabetes reduced the influence of an important metabolic determinant of circulating visfatin. Blood collection was performed under standardized fasting conditions, with samples from hemodialysis patients obtained before treatment following the longest interdialytic interval. Visfatin samples were stored under uniform conditions and analyzed together using the same assay, thereby limiting variation related to sampling time and between-run laboratory procedures.

## 5. Conclusions

In this non-diabetic study population, higher circulating visfatin concentrations were observed in patients receiving maintenance hemodialysis in unadjusted analyses; however, the overall between-group difference was attenuated and no longer statistically significant after adjustment for age, sex, hypertension, and body composition. Visfatin did not differ according to CAD status within the hemodialysis population, and its concentrations were similar across the CAD control, HD, and HD-CAD groups. The observed metabolic and biochemical correlations were exploratory and do not establish independent or causal relationships. Overall, our findings do not demonstrate an independent association between circulating visfatin and either hemodialysis status or clinically documented CAD. Prospective studies using matched groups, standardized assessment of coronary disease burden, larger sample sizes, and multivariable analyses are needed to clarify the clinical significance of visfatin in this population.

## Figures and Tables

**Table 1:** Demographic, clinical, and anthropometric characteristics.

Variable	HC (n = 40)	CAD-C (n = 28)	HD (n = 30)	HD-CAD (n = 32)	*p* Value
Age, years	47.5 (31–79)	58 (47–88)	59.5 (35–79)	62.5 (44–79)	<0.001
Female sex, n (%)	23 (57.5)	5 (17.9)	11 (36.7)	9 (28.1)	0.005
Hypertension, n (%)	6 (15.0)	12 (42.9)	19 (63.3)	25 (78.1)	<0.001
Systolic BP, mmHg	117.5 (90–160)	120 (90–150)	140 (90–160)	124.5 (90–160)	0.003
Diastolic BP, mmHg	80 (60–90)	80 (60–90)	80 (60–100)	79 (60–90)	0.646
BMI, kg/m^2^	28.35 (19.60–37.90)	25.70 (19.44–36.58)	24.66 (18.28–37.50)	24.48 (18.29–40.04)	0.003
Body fat, %	32.7 (19.3–60.0)	23.75 (12.3–49.8)	23.5 (10.8–60.0)	24.0 (14.8–60.0)	0.008
Body weight, kg	77.99 ± 15.43	75.71 ± 12.08	68.13 ± 14.11	68.12 ± 14.32	0.005
Waist circumference, cm	94.72 ± 12.25	94.25 ± 11.86	93.80 ± 11.63	94.71 ± 12.57	0.988
Dialysis duration, months	—	—	46.5 (12–228)	62 (13–216)	0.459

Continuous-variable distributions were assessed using the Shapiro–Wilk test. Normally distributed continuous variables are presented as mean ± standard deviation, non-normally distributed continuous variables as median (minimum–maximum), and categorical variables as n (%). *p* values represent overall comparisons among the four study groups, except for dialysis duration, which was compared between the two hemodialysis groups. HC, healthy controls; CAD-C, participants with coronary artery disease and preserved kidney function; HD, hemodialysis patients without clinically documented coronary artery disease; HD-CAD, hemodialysis patients with established coronary artery disease; BMI, body mass index; BP, blood pressure.

**Table 2:** Laboratory characteristics.

Variable	HC (n = 40)	CAD-C (n = 28)	HD (n = 30)	HD-CAD (n = 32)	*p* Value
Fasting glucose, mg/dL	88.5 (62–109)	87.5 (59–108)	92 (52–106)	98 (72–108)	<0.001
Insulin, μU/mL	8.1 (1.4–130.3)	8.9 (2.4–61.4)	12.15 (0.7–69.6)	20.30 (1.2–74.6)	<0.001
HOMA-IR	1.61 (0.25–30.53)	1.92 (0.45–14.23)	2.92 (0.08–17.16)	5.04 (0.22–19.32)	<0.001
Urea, mg/dL	28 (16–61)	31 (21–58)	128.5 (80–199)	130 (54–232)	<0.001
Creatinine, mg/dL	0.7 (0.6–1.1)	0.8 (0.6–1.2)	9.7 (4.5–14.9)	8.1 (2.4–13.0)	<0.001
Uric acid, mg/dL	4.25 (2.6–8.2)	5.85 (2.0–10.7)	5.9 (4.7–9.1)	6.1 (3.6–10.29)	<0.001
Calcium, mg/dL	9.45 (8.4–10.1)	9.4 (7.9–10.5)	9.25 (8.5–10.9)	8.7 (7.7–10.9)	<0.001
Phosphorus, mg/dL	3.2 (1.9–4.2)	3.1 (2.1–5.0)	4.9 (3.3–7.8)	5.0 (2.6–8.3)	<0.001
Albumin, g/dL	4.2 (3.6–4.9)	4.1 (2.9–4.9)	3.8 (3.3–4.4)	3.95 (3.1–4.5)	<0.001
Total cholesterol, mg/dL	201.5 (150–280)	176.5 (122–259)	153.5 (114–255)	184 (94–261)	<0.001
HDL cholesterol, mg/dL	45.5 (28–63)	39 (27–66)	34.5 (19–50)	30.5 (19–60)	<0.001
LDL cholesterol, mg/dL	129.5 (79–195)	108 (65–181)	88.4 (51–241)	97.6 (51–166)	<0.001
Triglycerides, mg/dL	116.5 (47–368)	118.5 (54–327)	114 (51–372)	195 (50.4–500)	<0.001
Hemoglobin, g/dL	14.1 (9.3–16.4)	13.8 (11.0–16.5)	11.3 (9.55–13.1)	11.8 (8.06–14.8)	<0.001
CRP, mg/dL	0.34 (0.30–1.48)	0.35 (0.30–7.26)	0.69 (0.10–2.08)	0.46 (0.10–2.30)	<0.001
PTH, pg/mL	50.65 (21.7–129.9)	69.35 (19.1–235.4)	443 (7.7–2431)	232.07 (3.12–1076.8)	<0.001
eKt/V	—	—	1.54 (1.01–2.38)	1.44 (1.03–2.17)	0.192
URR, %	—	—	72.36 (63.71–85.28)	74.88 (63.22–83.19)	0.667

Continuous-variable distributions were assessed using the Shapiro–Wilk test. All continuous variables presented in this table showed non-normal distributions and are therefore reported as median (minimum–maximum). *p* values represent overall comparisons among the four study groups. HC, healthy controls; CAD-C, participants with coronary artery disease and preserved kidney function; HD, hemodialysis patients without clinically documented coronary artery disease; HD-CAD, hemodialysis patients with established coronary artery disease; CRP, C-reactive protein; HDL, high-density lipoprotein; HOMA-IR, Homeostatic Model Assessment of Insulin Resistance; LDL, low-density lipoprotein; PTH, parathyroid hormone. eKt/V, equilibrated Kt/V; URR, urea reduction ratio. eKt/V and URR were assessed only in the hemodialysis groups; *p* values represent comparisons between the HD and HD-CAD groups.

**Table 3:** Serum visfatin concentrations and pairwise comparisons among the study groups.

Comparison	First Group Visfatin, ng/mL	Second Group Visfatin, ng/mL	Unadjusted *p*	Bonferroni-Adjusted *p*
HC (n = 40) vs. CAD-C (n = 28)	26.52 (9.10–47.18)	33.68 (13.25–55.30)	0.007	0.042
HC (n = 40) vs. HD (n = 30)	26.52 (9.10–47.18)	35.96 (11.62–46.61)	0.001	0.006
HC (n = 40) vs. HD-CAD (n = 32)	26.52 (9.10–47.18)	35.74 (9.21–61.64)	0.004	0.024
CAD-C (n = 28) vs. HD (n = 30)	33.68 (13.25–55.30)	35.96 (11.62–46.61)	0.773	1.000
CAD-C (n = 28) vs. HD-CAD (n = 32)	33.68 (13.25–55.30)	35.74 (9.21–61.64)	0.976	1.000
HD (n = 30) vs. HD-CAD (n = 32)	35.96 (11.62–46.61)	35.74 (9.21–61.64)	0.972	1.000

Visfatin concentrations showed a non-normal distribution according to the Shapiro–Wilk test and are presented as median (minimum–maximum). Pairwise comparisons were performed using the Mann–Whitney U test. Bonferroni correction was applied for six pairwise comparisons. Bold values indicate statistical significance after multiplicity correction. HC, healthy controls; CAD-C, participants with coronary artery disease and preserved kidney function; HD, hemodialysis patients without clinically documented coronary artery disease; HD-CAD, hemodialysis patients with established coronary artery disease.

**Table 4:** Adjusted serum visfatin concentrations according to study group.

Study Group	Adjusted Mean, ng/mL	95% CI
HC (n = 40)	28.40	24.84–31.95
CAD-C (n = 28)	33.60	29.61–37.59
HD (n = 30)	34.49	30.84–38.14
HD-CAD (n = 32)	33.75	30.00–37.51

Overall adjusted group effect: F(3, 122) = 2.054, *p* = 0.110, partial η^2^ = 0.048. Primary HD versus HD-CAD adjusted contrast: mean difference = 0.74 ng/mL, 95% CI −6.11 to 7.58, Bonferroni-adjusted *p* = 1.000. Sensitivity analysis: After replacing BMI with body fat percentage, the overall group effect remained non-significant, F(3, 122) = 2.537, *p* = 0.060, partial η^2^ = 0.059. Adjusted means are estimated marginal means from ANCOVA including study group, sex, and hypertension as fixed factors and age and BMI as covariates. Pairwise comparisons were Bonferroni-adjusted. A sensitivity model replaced BMI with body fat percentage. HC, healthy controls; CAD-C, participants with coronary artery disease and preserved kidney function; HD, hemodialysis patients without clinically documented coronary artery disease; HD-CAD, hemodialysis patients with established coronary artery disease; CI, confidence interval; BMI, body mass index.

**Table 5:** Correlations between serum visfatin concentration and selected clinical and biochemical variables.

Variable	HC (n = 40)	CAD-C (n = 28)	HD (n = 30)	HD-CAD (n = 32)
Age	−0.168 (0.300)	0.233 (0.234)	0.163 (0.390)	0.072 (0.696)
BMI	−0.062 (0.703)	0.046 (0.816)	0.135 (0.478)	0.172 (0.345)
Waist circumference	−0.039 (0.812)	0.066 (0.739)	0.263 (0.160)	0.036 (0.846)
Systolic BP	0.015 (0.925)	**0.496 (0.007)**	0.305 (0.102)	0.049 (0.790)
Fasting glucose	0.051 (0.753)	−0.034 (0.863)	0.065 (0.734)	0.204 (0.272)
Insulin	0.234 (0.146)	0.067 (0.733)	0.350 (0.068)	0.203 (0.274)
HOMA-IR	0.234 (0.146)	0.063 (0.749)	0.340 (0.077)	0.225 (0.223)
Urea	0.015 (0.928)	**0.469 (0.012)**	−0.185 (0.326)	0.045 (0.807)
Creatinine	−0.026 (0.874)	**0.381 (0.045)**	0.097 (0.610)	0.155 (0.396)
Uric acid	0.016 (0.921)	0.224 (0.252)	**0.394 (0.031)**	0.199 (0.275)
Total cholesterol	−0.048 (0.767)	−0.269 (0.166)	−0.021 (0.914)	0.108 (0.555)
Triglycerides	**−0.359 (0.023)**	−0.235 (0.230)	0.158 (0.413)	0.251 (0.166)
CRP	−0.260 (0.106)	0.170 (0.387)	0.046 (0.809)	0.080 (0.663)
PTH	0.163 (0.316)	0.213 (0.277)	−0.101 (0.596)	0.093 (0.612)

Values are presented as correlation coefficients, with *p* values in parentheses. Correlations were evaluated using Pearson or Spearman correlation analysis, as appropriate. Nominal associations with *p* < 0.05 are shown in bold. Analyses were exploratory, and *p* values were not adjusted for multiple testing. HC, healthy controls; CAD-C, participants with coronary artery disease and preserved kidney function; HD, hemodialysis; HD-CAD, hemodialysis patients with coronary artery disease; BMI, body mass index; BP, blood pressure; CRP, C-reactive protein; HOMA-IR, Homeostatic Model Assessment of Insulin Resistance; PTH, parathyroid hormone.

## Data Availability

The data are available from the corresponding author upon reasonable request. The data are not publicly available due to privacy and ethical restrictions.

## Abbreviations

The following abbreviations are used in this manuscript:

BMI	Body mass index
CAD	Coronary artery disease
CRP	C-reactive protein
ELISA	Enzyme-linked immunosorbent assay
HC	Healthy controls
HD	Hemodialysis
HDL-C	High-density lipoprotein cholesterol
HOMA-IR	Homeostatic model assessment of insulin resistance
LDL-C	Low-density lipoprotein cholesterol
PTH	Parathyroid hormone

## References

[1] Jankowski J., Floege J., Fliser D., Böhm M., Marx N. (2021). Cardiovascular disease in chronic kidney disease: Pathophysiological insights and therapeutic options. Circulation.

[2] Kidney Disease: Improving Global Outcomes (KDIGO) CKD Work Group (2024). KDIGO 2024 Clinical Practice Guideline for the Evaluation and Management of Chronic Kidney Disease. Kidney Int..

[3] Zoccali C., Mallamaci F., Adamczak M., de Oliveira R.B., Massy Z.A., Sarafidis P., Agarwal R., Mark P.B., Kotanko P., Ferro C.J. (2023). Cardiovascular complications in chronic kidney disease: A review from the European Renal and Cardiovascular Medicine Working Group of the European Renal Association. Cardiovasc. Res..

[4] Wang Y., Gao L. (2022). Inflammation and cardiovascular disease associated with hemodialysis for end-stage renal disease. Front. Pharmacol..

[5] Lo R., Narasaki Y., Lei S., Rhee C.M. (2023). Management of traditional risk factors for the development and progression of chronic kidney disease. Clin. Kidney J..

[6] Marx-Schütt K., Cherney D.Z.I., Jankowski J., Matsushita K., Nardone M., Marx N. (2025). Cardiovascular disease in chronic kidney disease. Eur. Heart J..

[7] Galley J.C., Singh S., Awata W.M.C., Alves J.V., Bruder-Nascimento T. (2022). Adipokines: Deciphering the cardiovascular signature of adipose tissue. Biochem. Pharmacol..

[8] Serrano E., Shenoy P., Martinez Cantarin M.P. (2023). Adipose tissue metabolic changes in chronic kidney disease. Immunometabolism.

[9] Semerena E., Nencioni A., Masternak K. (2023). Extracellular nicotinamide phosphoribosyltransferase: Role in disease pathophysiology and as a biomarker. Front. Immunol..

[10] Lim S.Y., Davidson S.M., Paramanathan A.J., Smith C.C.T., Yellon D.M., Hausenloy D.J. (2008). The novel adipocytokine visfatin exerts direct cardioprotective effects. J. Cell. Mol. Med..

[11] Yilmaz M.I., Saglam M., Carrero J.J., Qureshi A.R., Caglar K., Eyileten T., Sonmez A., Cakir E., Yenicesu M., Lindholm B. (2008). Serum visfatin concentration and endothelial dysfunction in chronic kidney disease. Nephrol. Dial. Transplant..

[12] Mu J., Feng B., Ye Z., Yuan F., Zeng W., Luo Z., Qi W. (2011). Visfatin is related to lipid dysregulation, endothelial dysfunction and atherosclerosis in patients with chronic kidney disease. J. Nephrol..

[13] Lu Y.C., Hsu C.C., Yu T.H., Wang C.P., Lu L.F., Hung W.C., Chiu C.A., Chung F.M., Lee Y.J., Tsai I.T. (2013). Association between visfatin levels and coronary artery disease in patients with chronic kidney disease. Iran. J. Kidney Dis..

[14] Nüsken K.D., Petrasch M., Rauh M., Stöhr W., Nüsken E., Schneider H., Dötsch J. (2009). Active visfatin is elevated in serum of maintenance haemodialysis patients and correlates inversely with circulating HDL cholesterol. Nephrol. Dial. Transplant..

[15] Axelsson J., Witasp A., Carrero J.J., Qureshi A.R., Suliman M.E., Heimbürger O., Bárány P., Lindholm B., Alvestrand A., Schalling M. (2007). Circulating levels of visfatin/pre-B-cell colony-enhancing factor 1 in relation to genotype, GFR, body composition, and survival in patients with CKD. Am. J. Kidney Dis..

[16] Carrero J.J., Witasp A., Stenvinkel P., Qureshi A.R., Heimbürger O., Bárány P., Suliman M.E., Anderstam B., Lindholm B., Nordfors L. (2010). Visfatin is increased in chronic kidney disease patients with poor appetite and correlates negatively with fasting serum amino acids and triglyceride levels. Nephrol. Dial. Transplant..

[17] Sabatino A., Cuppari L., Stenvinkel P., Lindholm B., Avesani C.M. (2021). Sarcopenia in chronic kidney disease: What have we learned so far?. J. Nephrol..

[18] Ikizler T.A., Burrowes J.D., Byham-Gray L.D., Campbell K.L., Carrero J.J., Chan W., Fouque D., Friedman A.N., Ghaddar S., Goldstein-Fuchs D.J. (2020). KDOQI Clinical Practice Guideline for Nutrition in CKD: 2020 Update. Am. J. Kidney Dis..

[19] Kato A., Odamaki M., Ishida J., Hishida A. (2009). Relationship between serum pre-B cell colony-enhancing factor/visfatin and atherosclerotic parameters in chronic hemodialysis patients. Am. J. Nephrol..

[20] Wang X., Yuan D., Shao F., Liu C., Zhang X., Li Z. (2026). Serum visfatin level and cardiac valve calcification in hemodialysis patients. Hemodial. Int..

[21] Hamim H.A., Satar D.A.J., Al-Musawi M.H.J., Shahriari-Khalaji M., Tavakoli M., Mirhaj M., Hamzah N.A. (2025). Role of visfatin in chronic kidney disease: Diagnostic potential and association with hemodialysis. Rep. Biochem. Mol. Biol..

[22] Jiao J., Wang X., Shao F., Yuan D., Li Z. (2024). A study on the correlation between serum visfatin and coronary artery calcification in maintenance hemodialysis patients. J. Cap. Med. Univ..

[23] Abdalla M.M.I. (2022). Role of visfatin in obesity-induced insulin resistance. World J. Clin. Cases.

[24] Niepolski L., Grzegorzewska A.E., Młot-Michalska M. (2010). Visfatin and endogenous secretory receptor for advanced glycation end-products in diabetic type 2 and non-diabetic patients undergoing intermittent hemodialysis. Int. Urol. Nephrol..

